# An Eight-CpG-based Methylation Classifier for Preoperative Discriminating Early and Advanced-Late Stage of Colorectal Cancer

**DOI:** 10.3389/fgene.2020.614160

**Published:** 2021-01-13

**Authors:** Ji Hu, Fu-ying Zhao, Bin Huang, Jing Ran, Mei-yuan Chen, Hai-lin Liu, You-song Deng, Xia Zhao, Xiao-fan Han

**Affiliations:** ^1^Department of General Surgery, The First People’s Hospital of Chongqing Liang Jiang New Area, Chongqing, China; ^2^Department of Microbiology, Army Medical University, Chongqing, China; ^3^Department of Medical Laboratory, The First People’s Hospital of Chongqing Liang Jiang New Area, Chongqing, China; ^4^Department of General Surgery, Daping Hospital, Army Medical University, Chongqing, China; ^5^Department of Pathology, The First People’s Hospital of Chongqing Liang Jiang New Area, Chongqing, China; ^6^Department of Clinical Pharmacy, The First People’s Hospital of Chongqing Liang Jiang New Area, Chongqing, China

**Keywords:** DNA methylation, CpG site, colorectal cancer, stage, classifier

## Abstract

**Aim:**

To develop and validate a CpG-based classifier for preoperative discrimination of early and advanced-late stage colorectal cancer (CRC).

**Methods:**

We identified an epigenetic signature based on methylation status of multiple CpG sites (CpGs) from 372 subjects in The Cancer Genome Atlas (TCGA) CRC cohort, and an external cohort (GSE48684) with 64 subjects by LASSO regression algorithm. A classifier derived from the methylation signature was used to establish a multivariable logistic regression model to predict the advanced-late stage of CRC. A nomogram was further developed by incorporating the classifier and some independent clinical risk factors, and its performance was evaluated by discrimination and calibration analysis. The prognostic value of the classifier was determined by survival analysis. Furthermore, the diagnostic performance of several CpGs in the methylation signature was evaluated.

**Results:**

The eight-CpG-based methylation signature discriminated early stage from advanced-late stage CRC, with a satisfactory AUC of more than 0.700 in both the training and validation sets. This methylation classifier was identified as an independent predictor for CRC staging. The nomogram showed favorable predictive power for preoperative staging, and the C-index reached 0.817 (95% CI: 0.753–0.881) and 0.817 (95% CI: 0.721–0.913) in another training set and validation set respectively, with good calibration. The patients stratified in the high-risk group by the methylation classifier had significantly worse survival outcome than those in the low-risk group. Combination diagnosis utilizing only four of the eight specific CpGs performed well, even in CRC patients with low CEA level or at early stage.

**Conclusions:**

Our classifier is a valuable predictive indicator that can supplement established methods for more accurate preoperative staging and also provides prognostic information for CRC patients. Besides, the combination of multiple CpGs has a high value in the diagnosis of CRC.

## Introduction

Colorectal cancer (CRC) is one of the most common malignancies, and ranks third in terms of both incidence and mortality rates. Around 1,47,950 new cases and 53,200 CRC-related deaths are projected for 2020 in the United States alone (Siegel et al., 2020). The incidence of CRC has increased by 38% between 2007 and 2017 (Global Burden of Disease Cancer Collaboration et al., 2019), and is therefore a critical public health concern.

Tumor node metastases (TNM) staging is currently the “gold standard” for tumor classification, and accurate diagnosis of the tumor stage provides valuable prognostic information for guiding treatment decisions (De Rosa et al., 2016). 5-year relative survival for CRC patients was 90⋅1% with localized stage, while it fell to 69⋅2% in patients with regional spread and to 11⋅7% in patients with distant metastasis (Brenner et al., 2014). For colon cancer and upper rectal cancer (defined as tumors arising above 10 cm of the anal verge), radical resection is the most common treatment for patients with stage I or those stage II without high-risk relapse. Preoperative lymph node status assessment and prediction contain instructive information for the surgical extent between stage I/II and stage III cases (lymph-node positive) (Hashiguchi et al., 2020). Postoperative adjuvant chemotherapy is recommended for all stage III CRC without contraindications after curative resection (Brenner et al., 2014). Except for adjuvant chemotherapy, preoperative neoadjuvant therapy, surgical resection and targeted therapies should be taken into consideration according to multidisciplinary team decisions for stage IV CRC (Diagnosis And Treatment Guidelines For Colorectal Cancer Working Group Csococ, 2019). Currently, computed tomography (CT) and magnetic resonance imaging (MRI) are commonly used for the preoperative assessment of CRC stages, although such imaging modalities have low accuracy due to some potential limitations (Tezcan et al., 2013; Kijima et al., 2014). Pathological stage is generally conducted after radical surgical resection rather than by preoperative biopsy. However, incomplete resection of tumor tissues or nodes missed by the surgeon may result in inaccurate pathological stage diagnosis (Mekenkamp et al., 2009). Therefore, it is essential to develop a reliable and efficient tool for preoperative CRC staging in order to devise the optimum personalized therapeutic strategy (De Rosa et al., 2016).

DNA methylation is an epigenetic modification that may regulate gene expression by altering the spatial conformation of DNA, and therefore controls a wide range of biological processes. Furthermore, studies increasingly show a close association between abnormal DNA methylation and pathological conditions, especially cancers (Portela and Esteller, 2010). Thus, aberrantly methylated CpGs are promising biomarkers for early diagnosis, molecular classification and prognosis in multiple cancers (Kaur et al., 2019). Previous studies mainly focused on identifying differentially methylated CpG sites with diagnostic and prognostic relevance in CRC. To the best of our knowledge, no study has investigated the predictive ability of preoperative staging using the methylation profiles of primary CRC samples. The aim of this study was to develop and validate a novel methylation classifier coupled with clinical features for preoperative classification of early stage and advanced-late stage in CRC patients.

## Materials and Methods

### Data Collection and Preprocessing

**FIGURE 1:**
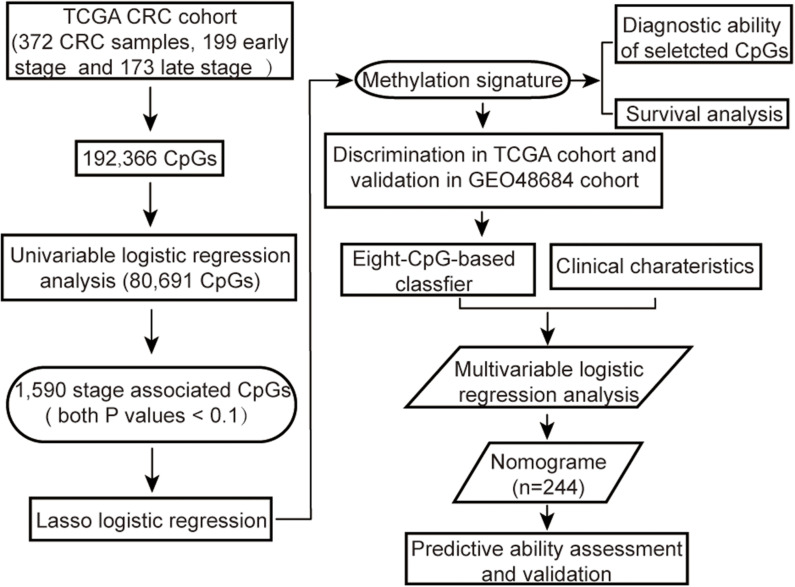
Flow chart showing the steps involved in systematical analysis.

The methylation array data of 443 samples from TCGA Colon and Rectal Cancer cohort (TCGA cohort) was downloaded by UCSC Cancer Browser^1^. In addition, the genomic methylation microarray dataset GSE48684 including 105 samples was downloaded from Gene Expression Omnibus (GEO, ^2^) (Luo et al., 2014). The clinicopathological characteristics and follow-up information were also extracted for all patients. The criteria for excluding samples were as follows: (a) non-primary tumors, (b) any history of neoadjuvant treatment, (c) unclear pathological stage information, or (d) with more than 5% missing values. In addition, for duplicated samples, only the sample with the highest average methylation levels was retained. Since both datasets had been generated using the Illumina Infinium HumanMethylation450 platform, the microarray probes were mapped onto the human genome coordinates using Illumina official annotation file derived from GEO GPL13534 platform. For each specimen, DNA methylation was quantified in terms of beta values for 485,577 individual CpGs. The CpGs probes i) with beta values undetectable in more than 5% of the specimens, ii) corresponding to cross-reactive probes in human reference genome (hg19) (Price et al., 2013) or single-nucleotide polymorphisms (SNPs) (Zhou et al., 2017), or iii) located on sex chromosomes (Chen et al., 2013), iv) or beta values with low variation among samples (the median absolute deviation < 25%) (Wang et al., 2012; Czamara et al., 2019) were removed from the analysis. The k-nearest neighbor (KNN) imputation algorithm implanted in the “DMwR” R package was used to estimate beta values of the other unidentified probes (Zhang et al., 2018). All methylation data were normalized, and then correction for batch effects was performed using “ComBat” function in R “sva” package before further analysis (Leek et al., 2012). The overall strategy was outlined in Figure 1.

### Candidate CpGs Screening

The methylation status of each CpG site in each sample was defined according to beta values, labeled as low methylation (beta value ≤ 0.2), intermediate methylation (0.2 < beta value < 0.6) and high methylation (beta value ≥ 0.6) (Novakovic et al., 2011; Yang et al., 2016). And then, those CpGs with three categories of methylation statuses simultaneously in all CRC samples were retained. Subsequently, all CRC cases with definite TNM stage information were then categorized into the early stage (stage I and stage II) and advanced-late stage (stage III and stage IV) groups in both cohorts. Finally, the predictive value of each CpG site methylation status for advanced-late stage CRC was determined by univariable logistic regression analysis, and those with both *P* values (for intermediate methylation versus low methylation; for high methylation versus low methylation) < 0.1 were retained.

### Features Selection and Methylation Signature Building

The TCGA CRC cohort was split into training set I and test set I in a 70/30 ratio, and the patients from GEO cohort (validation set I) were used for external validation. The most significant predictive CpGs were screened from the training set I using the least absolute shrinkage and selection operator method (LASSO) logistic regression algorithm (Friedman et al., 2010), and the candidate CpGs with penalty parameter tuning were selected by 10-fold cross-validation using the “glmnet” R package. The features with non-zero coefficients were identified based on the optimal lambda value, and considered the most significant predictive variables for further modeling. The methylation signature was developed on the basis of a methylation score that was calculated for each sample through a linear combination of selected CpGs weighted by their respective coefficients. The discriminating ability of the methylation signature was evaluated by plotting the receiver operation characteristic (ROC) curves in three cohorts. The areas under ROC (AUC) were calculated and their confidence intervals (CI) were estimated using bootstrap resampling method. Finally, the areas under the ROC curves in test set I and validation set I were compared by the bootstrap test.

### Construction and Validation for an Individualized Nomogram

An optimal methylation signature score cutoff was identified by the maximum Youden index based on the ROC curve, and a multiple-CpG-based classifier was constructed. The CRC cases in TCGA cohort were then categorized into the low- and high-risk groups according to the classifier. The samples with incomplete clinical information, including age, gender, personal history of polyps, preoperative carcinoembryonic antigen (CEA) and tumor location, etc. were further eliminated from TCGA cohort. Univariable regression analyses were initially performed to determine clinical risk factors associated with advanced-late stage in the remaining samples. Then, clinical factors with p ≤ 0.1 on univariable analyses along with the methylation classifier were tested in multivariable analyses in order to identify independent predictors of staging. Subsequently, we randomly divided the remaining cases into training set II and validation set II in a 70/30 ratio. A multivariable logistic regression model was constructed using those independent risk factors identified by multivariable analysis in training set II. Accordingly, a clinical epigenetic nomogram incorporating these predictors was then constructed based on this model.

The predictive performance of the nomogram was evaluated with respect to discrimination and calibration. Discrimination was evaluated with the area under the ROC curve in training set II and its confidence intervals were estimated employing bootstrap resampling method. Calibration curves were plotted with the Hosmer-Lemeshow goodness-of-fit test to assess calibration. For nomogram validation, we used 1,000 resampled bootstrapping method to relatively correct AUC in the development set. In validation set II, the nomogram was also validated by using AUC and calibration curve.

### Prognostic Values of the Classifier and Diagnostic Values of Multiple CpGs

Survival analysis was conducted on TGCA cohort after excluding cases with incomplete follow-up data or survival duration shorter than 30 days. Kaplan-Meier curves for overall survival (OS), disease-specific survival (DSS) and progression-free interval (PFI) were plotted for the risk subgroups, and compared with the log-rank test. In addition, the Mann-Whitney U test was used to analyze differences in the methylation levels of the above selected CpGs and the false discovery rate (FDR) was calculated to adjust the P values of each CpG site (Huang et al., 2017; Guo et al., 2018). To fully exploit the methylation status of those CpGs, a diagnostic model was constructed using LASSO logistic regression algorithm to distinguish tumors from normal tissues in a random 70% of samples selected from TCGA cohort (training set III), the performance of the model was estimated in the remaining 30% (test set III) and then externally validated using GEO cohort. Finally, ROC curve was applied to examine the diagnostic capability of the model in the cases with low CEA levels or at early tumor stage.

### Statistical Analysis

All statistical analyses were conducted using R software (version3.6.3; ^3^). Mann-Whitney U test was performed to compare beta values of the CpGs between CRC and normal controls. The Chi-square test or Fisher exact probability test was used for comparing categorical variables. The “glmnet” package was used for LASSO logistic regression analysis (Friedman et al., 2010), the “rms” package for logistic regression analysis and nomogram calibration, the “regplot” package for nomogram plots, and the “pROC” package for ROC plots (Robin et al., 2011). A two-sided *P* value less than 0.05 was considered statistically significant.

## Results

### Candidate Sites

A total of 372 CRC samples with well-defined pathological stages and 45 normal samples from TCGA cohort, and 64 CRC specimens with detailed stage information and 41 controls from the GEO cohort were included after applying the exclusion criteria. The TGCA CRC samples were randomly divided into the training set I (n = 260) and test set I (n = 112), and the GEO CRC cases were used as the validation set I (n = 64) as detailed in the methods. Furthermore, 192,366 CpGs were extracted from the DNA methylation dataset of TCGA COADREAD based on the screening criteria, of which 80,691 CpGs with three categories of methylation statuses in all CRC samples were examined in univariable logistic regression. Then, according to the previously described the criteria of *P* values (see “Materials and Methods”), 1590 CpGs remained strongly associated with the advanced-late stage.

### Methylation Signature Construction and Validation

**FIGURE 2:**
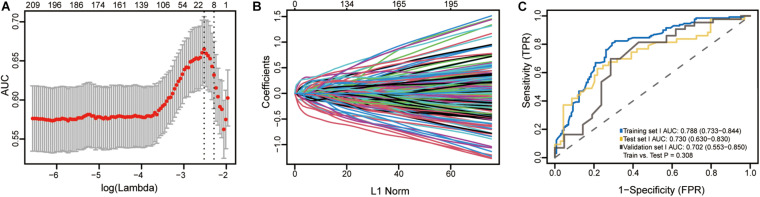
**(A)** Selection of tuning parameter (λ) in the LASSO model used 10-fold cross-validation in training set I by the 1 standard error of the minimum criteria (the 1-SE criteria). The AUC curve was plotted vs. log(λ). Dotted vertical lines were drawn at the optimal values using the minimum criteria and the 1-SE criteria. A λ value of 0.1013 with log (λ) of –2.290 was chosen. **(B)** LASSO coefficient profiles of the 1,590 CpGs. A coefficient profile plot was produced against the log (λ) sequence. **(C)** ROC curves showing discrimination ability of the methylation signature in training set I, test set I and validation set I.

After the initial screening of 1590 CpGs by LASSO logistic regression algorithm in the training set I, the optimal tuning parameter value of 0.1013 with log (λ) of −2.290 based on the 1 standard error of the minimum criteria (the 1-SE criteria) was selected using 10-fold cross-validation (Figure 2A). Accordingly, eight CpGs were identified as the most significantly correlated with CRC staging (Figure 2B), and the methylation score was calculated for each case as follows: (0.0104 × cg19922435 _*methylation status*_) − (0.0845 × cg10368049 _*methylation status*_) − (0.0901 × cg14931884 _*methylation status*_) − (0.0032 × cg23023937 _*methylation status*_) + (0.0841 × cg05817709 _*methylation status*_) − (0.0834 × cg27284627 _*methylation status*_) + (0.1529 × cg03124318 _*methylation status*_) + (0.0056 × cg19330334 _*methylation status*_). The annotations for these CpGs are shown in Supplementary Table S1. A methylation signature was then developed using the individual methylation scores, and its respective AUC values for the training set I, test set I and validation set I were 0.788 (95% CI: 0.733-0.844), 0.730 (95% CI: 0.630-0.830) and 0.702 (95% CI: 0.553-0.850). The bootstrap test further indicated similar discrimination performance of methylation signature between training set I and test set I (*P* = 0.308; Figure 2C). The clinical and pathological information are summarized in Supplementary Table S2.

### A Clinical Eepigenetic Nomogram Development and Corresponding Classification Performance

**FIGURE 3:**
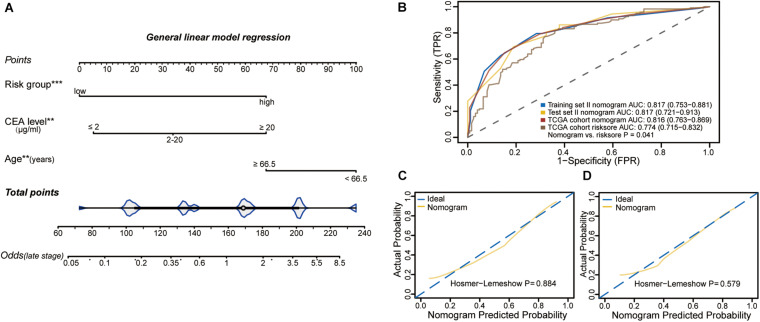
**(A)** The clinical epigenetic nomogram was developed in the training set II incorporating age, preoperative CEA levels and methylation classifier. **(B)** ROC curves showing the predictive performance of the nomogram in training set II and validation set II. Calibration curves of the nomogram with Hosmer-Lemeshow test in training set II **(C)** and validation set II **(D)**. The *x*-axis represents the predicted and y-axis the actual probability of late stage. The yellow solid line represents the performance of the nomogram, of which a closer fit to the dashed diagonal blue line indicates ideal prediction.

To construct an individualized nomogram, an eight-CpG-based classifier was developed with 0.496 as the optimal cutoff value of the methylation signature score. Next, according to this optimal cutoff value, 372 CRC cases were divided into the low-risk and high-risk groups. After exclusions, leaving 244 cases with essential clinical information for further analyses. Univariable logistic regression analyses identified age, CEA levels and the classifier as the potential risk factors (all *P* < 0.05). After adjustment for age and CEA levels, multivariable analysis indicated a 3.882-fold higher risk of advanced-late stage CRC in the high-risk compared to the low-risk group (95% CI: 2.510-6.164, *P* < 0.001, Table 1). In addition, age and CEA levels were also identified as independent factors for CRC staging (both *P* < 0.05). The 244 patients were randomly further split into training set II and validation set II, which were similar in all aspects (Supplementary Table S3). A multivariable logistic model was then established in training set II using the identified risk factors, and an inclusive nomogram was derived for preoperative staging in CRC patients (Figure 3A). The AUC of the nomogram for stage discrimination was 0.817 (95% CI: 0.753-0.881) in training set II (Figure 3B), which was corrected to 0.818 via bootstrapping validation (95% CI: 0.750-0.879), and 0.817 (95% CI: 0.721- 0.913) in validation set II. The bootstrap test indicated no significant differences between the two sets (*P* = 0.996). However, a statistically difference was observed for predictive performance between nomogram and methylation signature in 244 samples (*P* < 0.05; Figure 3B). Furthermore, the calibration curves of the nomogram showed good consistency between predicted and observed probability both in the training and validation cohorts, and the Hosmer-Lemeshow goodness-of-fit test also indicated statistical similarity (*P* = 0.884 and 0.579, respectively; Figures 3C, 3D). Taken together, the nomogram was fairly accurate in classifying CRC staging.

**TABLE 1:** Logistic regression analysis of clinical characteristics and methylation classifier.

**Characteristics**	**Univariable analysis**			**Multivariable analysis**		
	**OR**	**95% CI**	*P*-value*	**OR**	**95% CI**	*P*-value**
**Age (years)**						
≤66.5	1					
>66.5	0.546	0.377–0.785	0.001	0.500	0.321–0.768	0.002
**CEAlevels (ng/ml)**						
≤2	1					
2–20	7.250	3.220–20.864	<0.001	5.971	2.498–17.915	<0.001
≥20	2.368	1.368–4.582	0.004	1.943	1.068–3.897	0.040
**Methylation classifier**						
Low risk group	1					
High risk group	4.252	2.824–6.560	<0.001	3.882	2.510–6.164	<0.001

*, *P*-value was generated from univariable logistic regression analysis; **, *P*-value derived from multivariable logistic regression analysis. OR, odds ratio; CI, confidence interval; CEA, carcinoembryonic antigen.

### Additional Diagnostic and Prognostic Values

**FIGURE 4:**
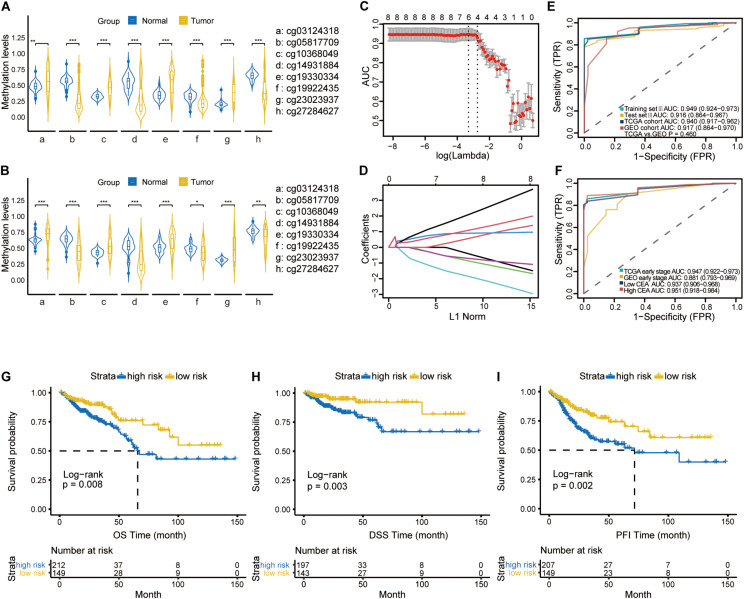
The violin plot diagrams depicting the differential methylation pattern of the CpGs in CRC versus normal samples using Mann-Whitney *U*-test in TCGA cohort **(A)** and GEO cohort **(B)**. **representing a *P*-value < 0.01; ***representing a *P*-value < 0.001. **(C)** 10-fold cross validation for tuning parameter selection via the 1-SE criteria. The optimal λ value of 0.0640 with log (λ) = –2.749 was selected. **(D)** LASSO coefficient profiles of the Eight specific CpGs in the screening process. **(E)** ROC curves demonstrating the ability of four-CpGs combination to identify CRC from normal samples in training set III, test set III, TCGA cohort and GEO cohort. **(F)** ROC curves indicating the differentiating ability of four-CpGs combination in CRC patients with low CEA levels or at early tumor stage from the normal samples in the TCGA and GEO cohorts, respectively. Kaplan-Meier curves showing OS **(G)**, DSS **(H)** and PFI **(I)** in the low- and high-risk subgroups. OS, overall survival; DSS, disease-specific survival; DFI, disease-free interval, and PFI, progression-free interval.

Furthermore, Violin plots for both TCGA (45 normal and 372 tumor samples, Figure 4A) and GEO (41 normal and 64 tumor samples, Figure 4B) datasets indicated that four of the CpGs signature had higher methylation levels (FDR-adjusted *P* < 0.01), while cg05817709, cg14931884, cg19922435 and cg27284627 had lower methylation levels in CRC compared to the normal samples (FDR-adjusted *P* < 0.001). To improve the stability and performance of diagnostic model and prevent overfitting, the LASSO logistic regression model was trained on the selected 8 CpGs. As a result, the optimal tuning parameter of 0.0640, with log(λ) = −2.749, obtained by performing 10-fold cross validation via the 1-SE criteria (Figure 4C), we identified another predictive methylation signature of four CpGs (Figure 4D). A diagnostic score for each sample based on individualized methylation status of the four CpGs was calculated as follows: Diagnostic score = (0.5077 × cg23023937_*methylation status*_) – (0.6461 × cg05817709_*methylation status*_) + (0.6302 × cg03124318_*methylation status*_) + (0.3378 × cg19330334 _*methylation status*_). The combination of these four sites showed high predictive accuracy for CRC, with a calculated AUC of 0.949 (95% CI: 0.924-0.973), 0.916 (95% CI: 0.864-0.967) and 0.940 (95% CI: 0.917-0.962) in training set III, test set III, and TCGA cohort, respectively (Figure 4E). The AUC of GEO cohort reached 0.917 (95% CI: 0.864-0.970; Figure 4E). Furthermore, the diagnostic ability of this model was also satisfactory in patients with CEA within the normal range (< 5ng/ml) (n = 235, AUC = 0.937, 95% CI: 0.906-0.968; Figure 4F). In patients at early stage of CRC, it achieved AUC of 0.947 (95% CI: 0.922-0.973) and 0.881 (95% CI: 0.793-0.969), respectively. Finally, Kaplan-Meier analysis showed that the OS (n = 361) and DSS (n = 340) of low-risk group were significantly higher than those of the high-risk group (both log-rank *P* < 0.01, Figures 4G, 4H). In addition, patients in the low risk group had significantly longer progression-free interval (PFI) compared to the high-risk group (log-rank *P* < 0.01; Figure 4I).

## Discussion

CRC is a global public health concern due to its high morbidity and mortality. The TNM stage of CRC remains an important determinant of therapy since it affects patient prognosis, recurrence and survival (Kawakami et al., 2015). Therefore, accurate stage classification is crucial for individualized treatment decisions at diagnosis, as well as improved outcomes. Preoperative staging currently relies on MRI and CT, instead of biopsy. However, the efficacy of imaging modalities is limited due to high costs, time and inaccuracy in T or N staging (Tezcan et al., 2013; Kijima et al., 2014). In addition, the established tumor markers CEA and CA19-9 also cannot accurately differentiate between CRC stages at diagnosis. Therefore, it is essential to build accurate predictive tools for preoperative staging. Studies have previously utilized differential -omics information to identify novel predictors associated with CRC development, such as nucleic acids, cytokines and proteins (de Wit et al., 2013; Abdulla et al., 2017; Nikolaou et al., 2018). However, small sample sizes, lack of further validation, and poor reproducibility in discriminating CRC stages have limited their potential clinical application.

CRC is characterized by significant molecular heterogeneity throughout its development (Koncina et al., 2020). Studies increasingly show that alterations in DNA methylation patterns are an important factor in CRC onset, progression and metastasis. As one of the earliest molecular events in cancer, aberrant DNA methylation is both stable and widespread (Klutstein et al., 2016; Lasseigne and Brooks, 2018). It is not unexpected that abnormal DNA methylation can serve as powerful biomarkers for diagnosis and prognosis, as well as promising targets for precision medicine in CRC (Liang and Weisenberger, 2017; Weisenberger et al., 2018). The bisulfite treatment-based methylation microarray (Illumina 450K Infinium) is commonly used for detecting cancer-related changes in individual CpGs and regions (Liang et al., 2019; Maros et al., 2020). In genome-wide methylation studies, the Illumina450k array covers more than 485,000 CpG sites across the entire genome, and allows high-throughput and relatively cost-effective bioinformatics analysis (Chen et al., 2016). To the best of our knowledge, the capacity of CRC methylation signature to differentiate between the early and late stages of cancer has not been explored so far. Therefore, the primary objective of this study was to develop an epigenetic signature with a minimum number of CpGs for CRC stage prediction.

Classification of cancer stages through epigenomics profiling is highly challenging compared to simply differentiating the normal tissues from malignant tissues (Kaur et al., 2019). Nevertheless, we systematically analyzed the DNA methylation data of CRC patients by multiple statistic methods, including LASSO logistic regression algorithm, univariable and multivariable logistic regression analysis, differential methylation analysis etc., which helped screen a set of CpGs related to tumor stage. Four of these CpGs – cg05817709, cg14931884, cg19922435 and cg27284627 – had lower methylation levels in CRC samples compared to normal tissues, and were mapped to the *RARRES3*, *DIP2C*, *LOC285419* and *NTM* genes respectively. The four remaining CpGs had higher methylation levels in CRC specimens, and were mapped to the *DPYSL4*, *COL1A2*, *USP30* and *IQGAP1* genes. As previously reported, most of the aforementioned genes are involved in tumor genesis and progression in multiple human malignancies, especially CRC (Jiang et al., 2005; Morales et al., 2014; Jin et al., 2015; Wang et al., 2015; Larsson et al., 2017; Ma et al., 2018). For instance, *COL1A2* encodes the pro-alpha2 chain of type I collagen, which is significantly associated with the pathological stage in CRC and correlates to patient OS and disease-free survival (DFS) (Ma et al., 2018; Zhou et al., 2018). In addition, the absence of *DIP2C* expression in CRC cells led to DNA methylation changes associated with gene expression and promoted cellular senescence and epithelial-mesenchymal transition (Larsson et al., 2017). *RARRES3* downregulation has been proven in multiple tumor types, including CRC tissues and re-expression of *RARRES3* exerted tumor-suppressive effects (Jiang et al., 2005; Morales et al., 2014; Wang et al., 2015). *IQGAP1* overexpression resulted in increased cell proliferation and migration via interaction with β-catenin in hepatocellular carcinoma cells (Jin et al., 2015).

Serum CEA level is the most accurate indicator of CRC recurrence following primary curative treatment (Duffy, 2001), and the positive association of elevated serum CEA with more advanced TNM stage and worse prognosis in CRC patients has been documented previously (Nicholson et al., 2015; Saito et al., 2016; Huang et al., 2018). Huang et al. reported preoperative CEA level ≥ 10 ng/mL as an independent predictive factor of OS (Huang et al., 2018). Likewise, Nicholson et al. recommended a CEA threshold of 10 μg/L for monitoring CRC recurrence following a systematic review of 52 studies (Nicholson et al., 2015). Not surprisingly therefore, patients with elevated serum CEA are more likely to be diagnosed at a more advanced stage. Indeed, patients both in the high CEA group (≥ 20 ng/mL) and in the median CEA group (2-20 ng/mL) in our cohort presented a statistically higher risk of late-stage disease compared to those with low CEA levels (≤ 2 ng/mL). Interestingly however, the younger CRC patients had higher scores in our nomogram. Andrew et al. analyzed possible risk factors for diagnosing late-stage CRC in a population-based study, and found that patients with early-onset CRC (< 50 years old) were more likely to be diagnosed at a later stage compared to those with late-onset CRC (≥ 50 years of age; OR 1.81, 95% CI: 1.27-2.58) (Andrew et al., 2018). This finding was also consistent with the report of Burnett-Hartman et al. (Burnett-Hartman et al., 2019). Compared to older patients with sporadic cancer, early-onset CRC has a higher incidence of adverse histological features (Chang et al., 2012), frequent absence of methylator phenotype and constitutively active oncogenic pathways (Kirzin et al., 2014), suggesting a more aggressive behavior (Meyer et al., 2016; Burnett-Hartman et al., 2019). Consistent with a previous study, we found that gender and race were not significantly related to CRC stage at the time of initial presentation (Andrew et al., 2018). In contrast to previous reports, however, we did not observe an association between history of polyps and lower risk of late-stage diagnosis (data not shown).

We established a predictive methylation signature using a panel of multiple CpGs to predict the risk of advanced-late stage CRC. Liang et al. had developed a 16-feature-based radiomics signature to preoperatively categorize CRC into stage I-II and III-IV, which was validated with an AUC of 0.708 (95% CI: 0.698-0.718) (Liang et al., 2016). Our methylation signature exhibits moderate predictive ability with AUC values greater than 0.700, which raises the possibility of combining two clinical predictors into a novel predictive model resulting in a greater accuracy. In addition, the classifier based on this methylation signature was an independent predictor of advanced-late stage CRC, and significantly improved the predictive ability of the nomogram.

The methylation signature-based predictive tool can supplement the currently established imaging modalities and biopsies in assessing CRC stages, and is particularly suitable for batch analysis of CRC samples. The methylation status based on beta values of the multiple CpGs can also provide additional diagnostic and prognostic information, and augment the clinical evidence in terms of selecting the most appropriate treatment strategy. However, our study has several limitations that ought to be considered. Firstly, absence of preoperative CEA levels and other clinical data in the GSE48684 dataset precluded a more rigid validation of the nomogram in an independent dataset. Secondly, insufficient preoperative indices, such as histological grade, family history and carbohydrate antigen 19-9 levels, limited other potential stage-related variables to be incorporated into our model. Thirdly, our nomogram still lacks experimental confirmation, and its reliability and reproducibility need to be verified by empirical methods. Fourthly, several prognostic models in CRC have been reported based on the methylation level of multiple sites, previously (Gündert et al., 2019; Wang et al., 2020). As an example, Melanie et al. developed a methylation-based classifier consisting of 20 CpG sites, which could improve the ability to predict survival in patients with non-metastatic CRC (Gündert et al., 2019). Regrettably, no overlap was found between the 8 CpGs and previously reported ones. Future analyses should further investigate whether our classifier might also serve as an independent predictor of survival, and whether it might be involved in a valuable prognosis model for CRC patients. In addition, even though our method requires a small amount of tissue, it is still invasive since it relies on biopsy samples. Finally, it is unclear whether the methylation changes in tumor tissues are consistent with those in the peripheral blood samples, and has to be clarified in future studies.

## Conclusion

We identified an eight-CpG-based methylation signature that classified CRC stages with considerable accuracy and then derivatized a methylation classifier. The nomogram incorporating the CpG classifier and clinical features had a satisfactory predictive power, and can potentially augment imaging and biopsy findings for accurate preoperative staging and expedited therapy. In addition, the combination of four CpGs showed a good diagnostic value in CRC patients, even in those with low serum CEA level or at early tumor stage, indicating a novel biomarker for early CRC diagnosis. Our strategy can be further applied to identify methylation signatures for lymphatic infiltration or distant metastasis of CRC.

## Data Availability Statement

Publicly available datasets were analyzed in this study. This data can be found here: https://xena.ucsc.edu/, https://www.ncbi.nlm.nih.gov/geo/query/acc.cgi?acc=GSE48684.

## Author Contributions

XZ and X-fH designed the study and revised the manuscript. JH and F-yZ collected and analyzed the data, screened candidate variables, built prediction models, and wrote the manuscript. BH, JR, M-yC, and H-lL helped in the interpretation, analysis of the data and models validation. All authors have read and approved the final manuscript and therefore, have full access to all the data in the study and take responsibility for the integrity and security of the data.

## Conflict of Interest

The authors declare that the research was conducted in the absence of any commercial or financial relationships that could be construed as a potential conflict of interest.

## Footnotes

1 https://xena.ucsc.edu/

2 https://www.ncbi.nlm.nih.gov/geo/

3 http://www.r-project.org
